# *In Situ* Pseudopotentials for Electronic Structure Theory

**DOI:** 10.1021/acs.jpcc.1c04791

**Published:** 2021-06-29

**Authors:** Kristofer Björnson, John Michael Wills, Mebarek Alouani, Oscar Grånäs, Patrik Thunström, Chin Shen Ong, Olle Eriksson

**Affiliations:** † Department of Physics and Astronomy, Uppsala University, Box 516, SE-75120 Uppsala, Sweden; ‡ Los Alamos National Laboratory, Los Alamos, New Mexico 87545, United States; ¶ Institut de Physique et Chimie des Matériaux de Strasbourg, Université de Strasbourg, UMR 7504 CNRS-UNISTRA, Strasbourg, France; § School of Science and Technology, Örebro University, Fakultetsgatan 1, SE-701 82 Örebro, Sweden

## Abstract

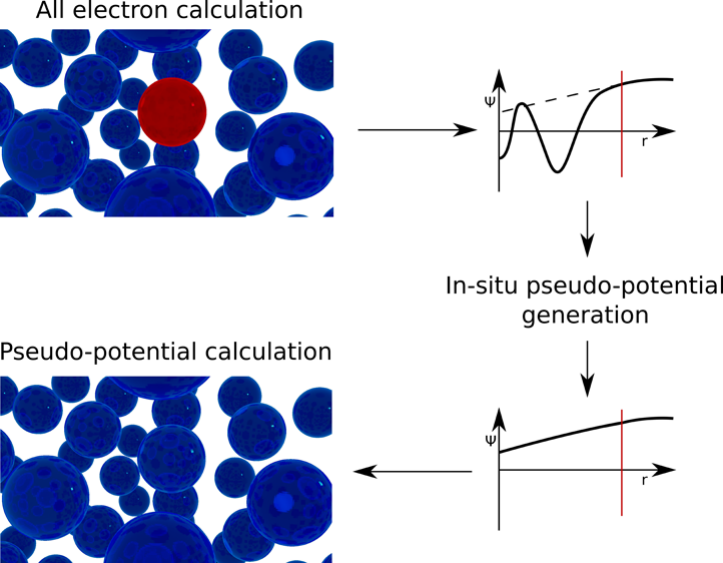

We present a general method of constructing *in situ* pseodopotentials from first-principles, all-electron, and full-potential electronic structure calculations of a solid. The method is applied to bcc Na, at low-temperature equilibrium volume. The essential steps of the method involve (i) calculating an all-electron Kohn–Sham eigenstate, (ii) replacing the oscillating part of the wave function (inside the muffin-tin spheres) of this state, with a smooth function, (iii) representing the smooth wave function in a Fourier series, and (iv) inverting the Kohn–Sham equation, to extract the pseudopotential that produces the state generated in steps i–iii. It is shown that an *in situ* pseudopotential can reproduce an all-electron full-potential eigenvalue up to the sixth significant digit. A comparison of the all-electron theory, *in situ* pseudopotential theory, and the standard nonlocal pseudopotential theory demonstrates good agreement, e.g., in the energy dispersion of the 3s band state of bcc Na.

## 1 Introduction

The electronic structure of solids within the density functional theory (DFT) has been solved by a variety of methods, such as the linear combination of atomic orbitals (LCAO),^1^ the Korringa–Kohn–Rostoker (KKR) Green function method,^2,3^ the all-electron linear muffin-tin orbitals (LMTO) and the linear augmented plane waves (LAPW),^4−6^ and the pseudopotential method.^7−11^ One key difference between the all-electron and the pseudopotential methods is in their treatments of core electrons. Whereas in all-electron methods the core electrons are explicitly included in the calculations, pseudopotential methods replace the potentials from the core states in the one-electron Schrödinger (or Kohn–Sham) equation with an effective smooth potential, known as the pseudopotential. This allows the pseudopotential methods to replace the valence states with smooth pseudowave functions, which have fewer nodes than the all-electron wave functions but the same eigenenergies.

This approach has its roots in the ideas of Fermi and Hellman more than 80 years ago,^12,13^ with the rigorous formulation of the theory for a solid taking place 20 years after that.^14−17^ A practical method of predicting energy band structures for semiconductors was only achieved upon the development of the empirical pseudopotential method (EPM),^8,9^ in which the pseudopotential is fitted to experimental band structures, establishing the validity of the energy band concept for solids in general. Nonetheless, empirical pseudopotentials are not always transferable between systems of different chemical environments since their suitability for a particular system depends on the similarity of that environment to the experimental environment to which the empirical pseudopotential was fitted.^9,11^ Consequently, the use of pseudopotentials constructed from first-principles, i.e., *ab initio* pseudopotentials, has become widespread in modern-day electronic structure research. *Ab initio* pseudopotentials can be norm-conserving^18,19^ or ultrasoft.^20,21^ In the projector augmented wave (PAW) approach,^22,23^ pseudopotential operators are also used, but information regarding the nodal structure of the all-electron wave functions in the core region is retained. Several excellent papers and reviews have already discussed many necessary details of the *ab initio* pseudopotential approach.^10,24,25^ Here, we will only reiterate some of the pertinent points.

The first step in constructing an *ab initio* pseudopotential usually involves solving the Kohn–Sham equations for a free isolated atom to obtain its all-electron eigenvalues and wave functions. In the second step, one constructs a smooth pseudowave function, which has a radial component that is identical with the radial component of the all-electron wave function outside a chosen cutoff radius, *r*_*c*_, but is smooth and nodeless inside this radius. Finally, one asks if there exists a potential (i.e., a pseudopotential) that when used together with the kinetic-energy operator to construct the Kohn–Sham Hamiltonian, produces this pseudowave function as its eigenstate upon diagonalization, while retaining the same all-electron energy eigenvalue of the free atom. This is achieved by inverting the Schrödinger equation. The pseudopotential approach takes advantage of the fact that the core electrons do not play an important role in the formation of chemical bonds between atoms.^13^ If all chemical bond formations, electron hopping, and effects leading to band-energy dispersion in a solid take place outside *r*_*c*_, one can replace the all-electron potential around each atom position of a solid with a lattice of pseudopotentials. Clearly, this can be extended to molecular and cluster entities.

**Figure 1:**
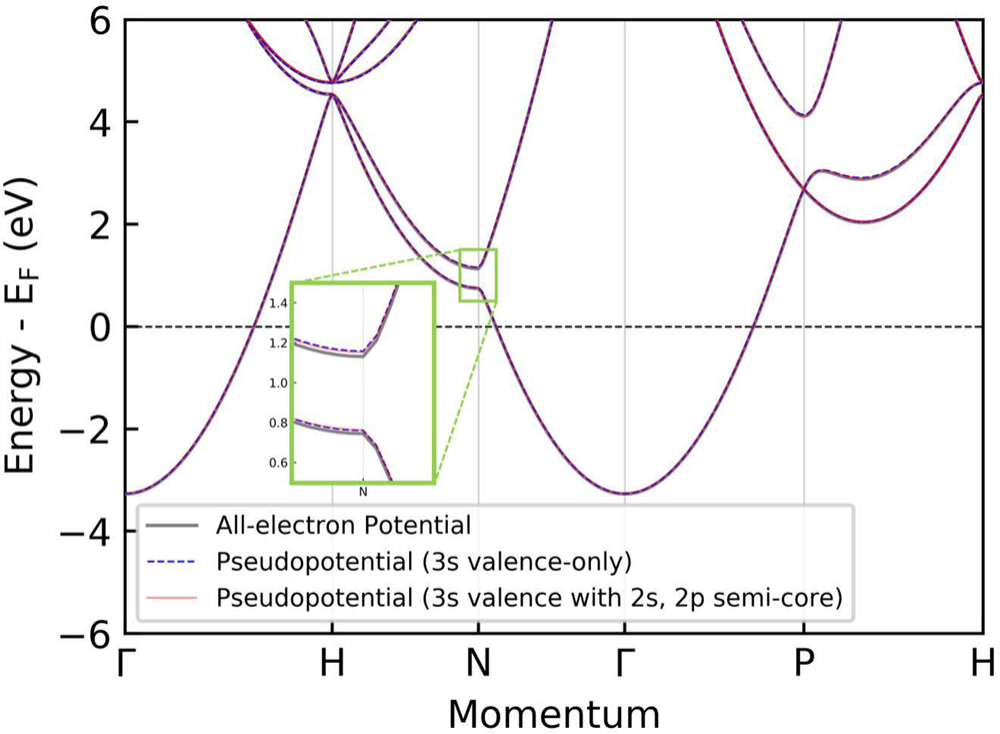
Comparison of the DFT energy dispersion for bcc Na within the local density approximation (LDA) using the experimental equilibrium unit-cell volume, calculated using the full-potential all-electron electronic structure method with RSPt (gray) and the pseudopotential method with Quantum Espresso. Two atomic pseudopotentials are used, with (red solid line) and without (blue dashed line) the semicore 2s state. The energy levels are plotted relative to the Fermi level (*E*_*F*_). In the inset a magnified section of the band dispersion around the *N*-point is shown, in order to highlight the magnitude of the differences between the methods. These calculations use the same convergence parameters as in Figure 2.

In a recent study,^26^*ab initio* pseudopotential electronic structure results were found to be in good agreement with those computed using all-electron theory. Indeed, the comparison in ref (26) was made for 71 elements, and the agreement of the all-electron and pseudopotential results, for materials with very different types of chemical bonding, supports the use of the computationally more efficient pseudopotential method. An example of comparison of the energy dispersion calculated using the all-electron and the standard nonlocal pseudopotential methods is shown in Figure 1. The figure illustrates the energy bands of bcc Na at the experimental unit-cell volume *V*_0_(27) (corresponding to the lattice constant = 4.225 Å). It is clear from the figure that pseudopotential and all-electron electronic structure theories can produce very similar band dispersion when the pseudopotential is properly constructed.

For a pseudopotential to have general applicability, it is important that it is transferable^28,29^ to different chemical environments, e.g., works for a molecule or a material that forms covalent or ionic bonds, under ambient or high pressure or even for a crystal surface. The transferability of a pseudopotential characterizes the accuracy with which the pseudopotential reproduces the effect of the all-electron potential in different chemical environments. One way to test for the transferability of a pseudopotential to a different chemical environment is by comparing the calculated Kohn–Sham energy eigenvalues with the self-consistent all-electron results.^28^ This raises the question of whether we can apply the normal protocols for generating pseudopotentials, but instead of using the atomic state as reference electronic configuration, we use an environment that more closely resembles the one where the pseudopotential is supposed to be used, be it in a solid or molecular state. We will refer to the standard pseudopotential derived from the free isolated atom as an atomic pseudopotential. In this work, we also use the solid state as a reference configuration, and we refer to such pseudopotentials as *in situ* pseudopotentals. We will demonstrate as a proof-of-concept in this paper that these pseudopotentials can reproduce all-electron results to very high accuracy.

The advantage of an *in situ* pseudopotental is that it is tailored to the specific chemical environment of the material (e.g., under high compression), and as a result, it can in general be used as an expedient, accurate, and computationally inexpensive tool to analyze electronic structures of complex systems, e.g., as discussed in ref (30). Therefore, the concern whether or not a pseudopotential is transferable or not may be eliminated, as we only need a recipe of how to generate it for a specific material. Even though the plane wave basis set used in pseudopotential theory is much larger than that of any all electron method, it offers the following advantages: (1) the rapidity of computation for each matrix element, (2) the ease of calculating Hellman–Feynman forces to obtain geometry optimization and to be able to compute the phonon spectra, (3) the simplicity of computing matrix elements of any perturbation to the Hamiltonian, including oscillator strength matrix elements, or many-body self-energy matrix elements, (4) the use of fast Fourier transform to speed up the calculation, and (5) its natural connection to self-energy methods, such as the *GW* approximation, for improved calculations of the energy band gap. In addition, the use of the solid-state environment to generate *in situ* pseudopotentials is motivated by the fact that the scattering properties of a pseudopotential constructed for an isolated atom might be different from those of the same atom placed in a material. This is particularly of concern when the environment in the solid is drastically different from that of an atom, e.g., when neighboring atoms in an ionic bonded material cause large charge transfer effects that affect the multiple scattering properties. Similarly, it can be troublesome to use an atomic generated pseudopotential evaluated at ambient conditions for a solid state calculation of the electronic structure of a material under extreme compression.

In this paper, we outline the critical steps to generate *in situ* pseudopotentials, and as example, we calculate for bcc Na the band dispersion using an *in situ* pseudopotential generated from an all-electron reference state obtained from the full-potential linear muffin-tin orbitals method (RSPt software^6^). This result is then compared with the band structure obtained from two different atomic (i.e., generated from an atomic reference state), scalar-relativistic and norm-conserving, pseudopotentials using the Quantum Espresso software.^31^ The main difference is that the first atomic pseudopotential (i) contains only the valence 3s state of Na (i.e., valence-only) while the second (ii) contains not only the valence 3s state but also the 2s and 2p semicore states. The former^32^ pseudopotential is a Troullier–Martins^33^ pseudopotential generated using FHI98PP^34^ and includes nonlinear core correction.^35^ It has only one Kleinman–Bylander–Vanderbilt^20,25^ projector per angular channel. The latter pseudopotential is an optimized norm-conserving Vanderbilt pseudopotential (ONCVPSP)^36^ obtained from the PSEUDODOJO project.^37^ It does not use nonlinear core correction and has two projectors per angular channel. All pseudopotential calculations with Quantum Espresso use a kinetic-energy cutoff of 100 Ry for the plane wave basis expansion, a **k**-grid of 24 × 24 × 24 points and LDA^38^ for the self-consistent DFT calculation.

## 2 Method

Simply described, the method to generate *in situ* pseudopotentials can be divided into three distinct steps: (1) Calculate the eigenstates of the Kohn–Sham equations of the solid using an all-electron method. (2) Construct the pseudowave function by modifying the corresponding valence state to remove any nodes in the core region while exactly preserving the wave function in the interstitial region. (3) Generate a pseudopotential that gives rise to this pseudowave function. We begin by describing step 3 in Section 2.1 before moving on to the more technical details of step 2 in Section 2.2.

### 2.1 The Inverse Pseudopotential Problem

For the purpose of this section, we first assume that the pseudowave function Ψ̃_**k***n*_ and its energy eigenvalue ϵ̃_**k***n*_ for each **k** point in the Brillouin zone and band *n* are already known. Next, we consider the eigenvalue equation
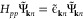
1where **k** is crystal momentum restricted to the first Brillouin zone, *n* a band index for the given **k**, and
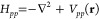
2
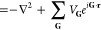
3
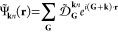
4

Here, **G** runs over the reciprocal lattice vectors, and *V*_**G**_ and  are, respectively, the plane wave expansion coefficients for the pseudopotential *V*_*pp*_ and pseudowave function Ψ̃_**k***n*_. The Ψ̃_**k***n*_ is known as the pseudowave function as it is the solution of a Hamiltonian involving a pseudopotential. In eqs 2 and 3 we have used Rydberg atomic units (au). If we multiply eq 1 by e^–^*^i^*^(^**^G^**^^′^+^*^k^*^)·^**^r^** and integrate over **r**, we obtain the expression
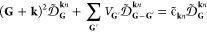
5

In practice, the sum over **G**′ in the plane-wave expansion of the pseudopotential and pseudowave function is truncated for a finite number (*N*) of coefficients. Therefore, for a given eigenstate eq 5 corresponds to *N* linear equations with *N* unknowns, which in matrix form can be written as (note that **k** and *n* are state labels and not matrix indices)
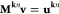
6where
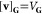
7
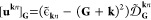
8
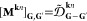
9

The matrix **M**^**k***n*^ is a blocked Toeplitz matrix. The linear system of equations in eq 6 can therefore be solved by a blocked Levinson algorithm, which formally produce the pseudopotential
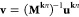
10

While this pseudopotential is only constructed to exactly reproduce the eigenvalue ϵ̃_**k***n*_ for a particular **k**, we will see in Section 3 that the procedure gives a pseudopotential that gives satisfactory results for eigenvalues throughout the Brillouin zone, i.e., also for eigenvalues calculated at **k**′ ≠ **k**.

### 2.2 Choice of Wave Function and Practical Implementation

While the method described in Section 2.1 is straightforward, the difficulty lies in generating an appropriate wave function Ψ̃_**k***n*_ to use as input. In principle, one can calculate it by first solving an all-electron electronic structure problem, thereby obtaining the true energy eigenvalue and wave function. Hence, by finding a solution to
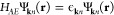
11where *H*_*AE*_ is the all-electron Hamiltonian, one could in principle solve eqs 7–10, by setting Ψ̃_**k***n*_ equal to the true all-electron wave function Ψ_**k***n*_ and by identifying ϵ̃_**k***n*_ with ϵ_**k***n*_. In this way, one is guaranteed that the pseudopotential that comes out of eq 10 gives the same eigenvalue and wave function as the all-electron Hamiltonian. A pseudopotential generated at one particular **k** point, e.g., the Γ-point, can then be used in eqs 1–3, to calculate eigenvalues and eigenstates throughout the Brillouin zone. One can also envision using this pseudopotential for other similar conditions, e.g., a crystal at compressed volumes compared to the condition where the pseudopotential is originally calculated from. In this approach, the valence states generated by an all-electron calculation are expected to have nodes in the core region, which will require a very large number of the basis vectors to converge the calculation if the plane-wave basis set is used (eq 4). A solution to this problem is to replace the fast oscillating part of Ψ_**k***n*_, that is primarily close to an atomic nucleus, with a smooth pseudowave function, Ψ̃_**k***n*_ that is nodeless while still keeping ϵ̃_**k***n*_ = ϵ_**k***n*_. This is the usual way of pseudopotential theory, albeit we propose here to do it using its native solid state as the reference electronic configuration and not the free atom.

The description that follows aims at describing how a smooth, node-less pseudowave function can be evaluated from an all-electron wave function that is obtained from a full-potential linear muffin-tin orbital method, as implemented in the RSPt package.^6^ We start with the general approach of writing the all-electron wave function as a linear combination of known basis functions, namely, the linear muffin-tin orbitals (LMTOs):
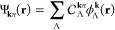
12Here ϕ_Λ_^**k**^ are the LMTOs introduced by Andersen,^4^ where the Λ index groups many indices, such as the tail energy of the basis function, angular momenta, and the type of atomic species. We emphasize that **k***n* are state labels, not indices. The LMTOs are defined with respect to two regions: the muffin-tin and the interstitial regions. In the latter, the LMTO basis function is either a Hankel or a Neumann function, depending on choice of kinetic energy of this basis function. For practical reasons, in the RSPt package,^6^ the wave function in the interstitial region is calculated as an exact Fourier series. This is done by extending a Hankel or Neumann function from the interstitial region into the muffin-tin sphere with an analytic smooth function, for fast converged Fourier series expansion. This means that the interstitial basis function is defined over all space, and matrix elements of, e.g., the Hamiltonian or the Bloch wave function overlap are truncated inside the muffin-tins using a step function.

Following ref (6), we define a pseudo basis function ϕ̃_Λ_^**k**^ as the Fourier-transformed Hankel or Neumann function that in the interstitial region is identical with the all-electron LMTO basis function:
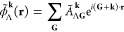
13where *Ã*_Λ**G**_^**k**^ are the Fourier coefficients of this basis function. This function can now be used for the expression of the Fourier series in eqs 1–4 that should be defined over all space, i.e., including the muffin-tin region. We start by expressing the interstitial all-electron wave function in eq 11 in terms of the Fourier series, see eq 13. This can be done by replacing ϕ_Λ_^**k***n*^ in eq 12 with ϕ̃_Λ_^**k***n*^. By construction, this replacement does not influence the wave function in the interstitial region. However, it drastically modifies the wave function in the muffin-tin region since the part of the muffin-tin orbital that is defined in the muffin-tin sphere, where in general the radial component has many nodes, is replaced by a smooth function. For this reason, we distinguish the true wave function given by eq 12 from a pseudowave function
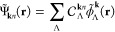
14

Notice that the expansion coefficients in eqs 12 and 14 and  should be the same. Writing out explicitly the form of ϕ̃_Λ_^**k***n*^, we can express the pseudowave function as
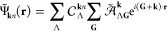
15

Using this equation, the coefficients  of eq 4 will be given by
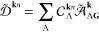
16

The form given by the latter equation should be used in the pseudowave function given in eq 4 to calculate the corresponding pseudopotential by following eqs 6–10. A practical way to evaluate a pseudopotential with this method is to first perform a normal all-electron calculation to obtain  coefficients (and the eigenvalue ϵ_**k***n*_). In this process, the Fourier coefficients of the pseudobasis function are kept (from eqs 13 and 16), which enables an evaluation of eq 4. The so-obtained pseudowave function and eigenvalue are used in eqs 6–10, to obtain the required pseudopotential.

Although the description above seems straightforward, we note that these modifications are done in the full-potential method of ref (6), independent of whether a pseudopotential is to be extracted or not. They are in line with the aims of the pseudopotential approach, but they are strictly speaking related to the computational benefits associated with having fewer coefficients in the Fourier expansion. To be useful for a pseudopotential approach, we also need to ensure that low-lying “ghost states” do not appear. To understand the “ghost states” problem, consider applying eq 10 immediately to the unmodified valence state. By construction, the resulting pseudopotential gives rise to a Hamiltonian that contains this eigenstate. However, the full Hilbert space also contains smoother states, and these tend to have lower energy. This is not surprising since states with lower energy do exist in the original problem, namely the core states. The purpose of the pseudopotential approach is to generate an effective Hamiltonian for which the valence states are the low energy states, and it is therefore essential to remove radial nodes in the wave function. While the procedure outlined above does reduce the number of radial nodes, it is not constructed to guarantee an absence of nodes. It also does not guarantee norm conservation. In this work, we are focused on constructing norm-conserving pseudopotentials even though this constraint of norm-conservation can be relaxed in future works similar to that in the ultrasoft-pseudopotential method^20,21^ or the PAW method.^22,23^ This of course comes at the expense of a more complex mathematical representation compared to the simple representation of norm-conserving pseudopotential method^33,39^).

For these reasons we modified the pseudowave function further to make it nodeless and ensure norm conservation. It is the aggregate of these modifications to the pseudowave function that are compensated for through the pseudopotential. Only by modifying the wave function in the core region is it possible to preserve the chemical properties that emerge from the pseudopotential. That is, chemical bonding is mainly determined by the wave function overlap in the interstitial region between atoms. The expression we arrived at for the nodeless and normalized pseudowave function is
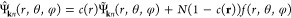
17where *f*(*r*,θ,ϕ) is a smooth function with the same angular dependence as Ψ̃_**k***n*_(*r*,θ,φ) and *c*(**r**) smoothly interpolates between Ψ̃^**k***n*^(*r*,θ,φ) in the interstitial region and *f*(*r*,θ,φ) in the muffin-tin region. For convenience, we have also factored out a constant *N* from *f*(*r*,θ,φ) that will be used to ensure that the pseudowave function is normalized. Note that eq 17 is general, in the sense that it can be applied to an all-electron valence state, although for technical reasons we applied it to the pseudowave function obtained from eq 16, Ψ̃^**k***n*^.

In this paper, we provide a proof-of-principle demonstration of the method for the energy dispersion of the Na 3s band, and in future work, we hope to generalize the method to compute the electronic structure of any material. Adapted for this state we choose
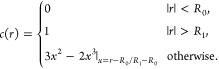
18

**Figure 2:**
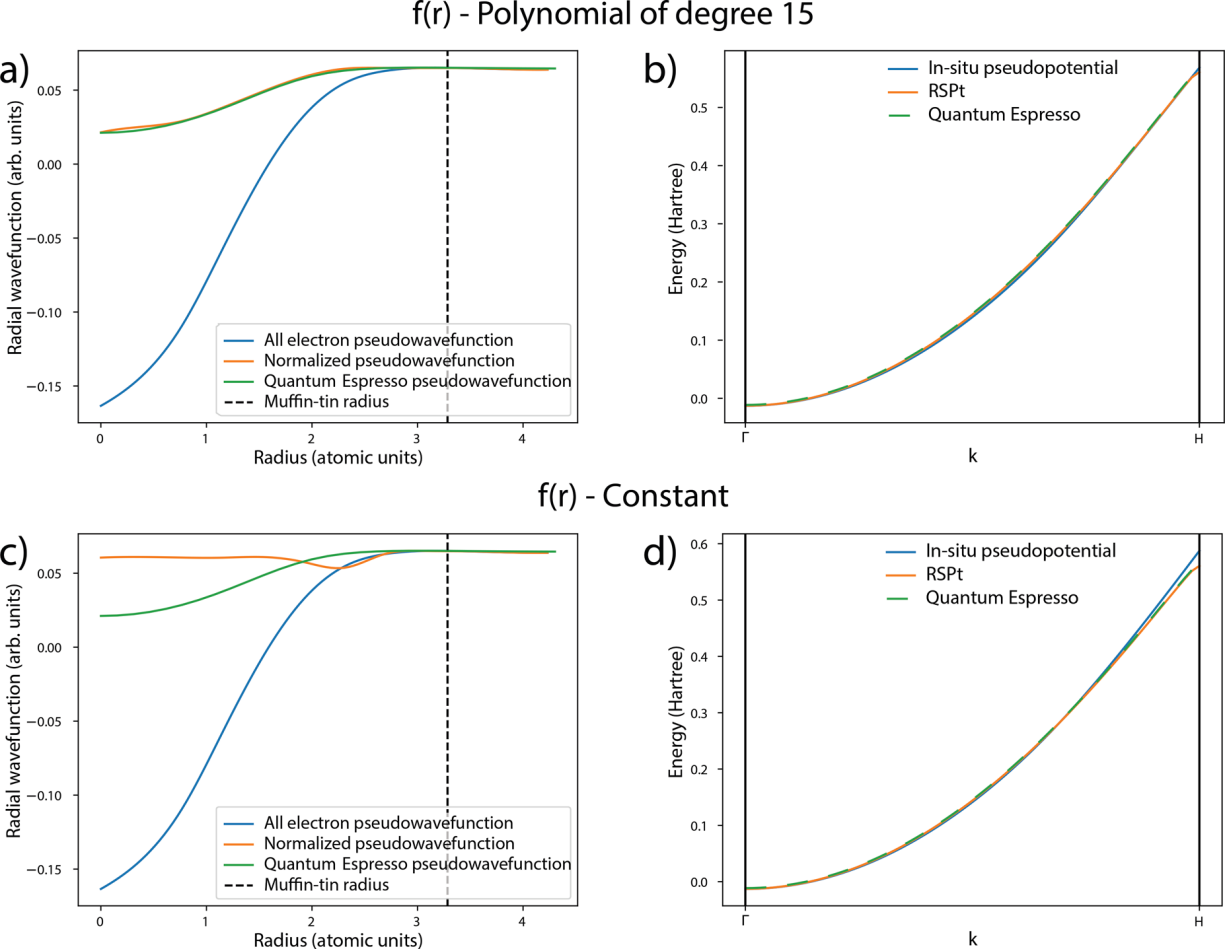
(a and c) Calculated radial component of the pseudowave function Ψ̃_**k***n*_(*r*,θ,φ) (blue, for definition see text), calculated radial part of the modified pseudowave function Ψ̂_**k***n*_(*r*,θ,φ) (orange, for definition see text), and radial pseudowave function calculated with Quantum Espresso using a valence-only atomic pseudopotential (green). (b and d) Calculated energy dispersion of the valence band states of bcc Na along the Γ – *H* high symmetry direction of the first Brillouin zone. Three types of methods are used to calculate the energy bands: the all-electron full potential method (orange), the *in situ* pseudopotential method as described of this paper (blue), and the standard pseudopotential method using a valence-only atomic pseudopotential (green). Panels a and b show results for the choice of *f*(*r*) being a polynomial of degree 15 that is least-squares fitted to the radial pseudowave function calculated with Quantum Espresso using a valence-only pseudopotential (using *R*_0_ = 0.75*R*_*MT*_ and *R*_1_ = 0.9*R*_*MT*_). Panels c and d show results for a choice of *f*(*r*) = 1 (using *R*_0_ = 0.55*R*_*MT*_, and *R*_1_ = 0.75*R*_*MT*_. The muffin-tin radius *R*_*MT*_ = 3.285 au is denoted using dashed lines in panels a and c). In all these calculations, a 11 × 11 × 11 mesh is used for the Fourier expansion of the pseudopotential.

The node-free and normalized pseudowave function is then defined with a suitable choice of *f*(*r*,θ,ϕ) in eq 17. We will return to appropriate choices of this function below. First we remark that, in eq 18, *R*_0_ and *R*_1_ are chosen so as to obtain a smooth interpolation without nodes. In general, *R*_0_ should be larger than the radius of the outermost radial node, and we have *R*_0_ < *R*_1_ < *R*_*MT*_, where *R*_*MT*_ is the muffin-tin radius. Returning now to the choice of *f*(*r*,θ,ϕ), we have in the numerical examples presented below focused on the valence state of Na, which is dominated by the 3s state. This function has no angular component, and it is sufficient to use *f*(*r*,θ,ϕ) = *f*(*r*). We have made two choices for *f*(*r*): a constant value of 1 and a polynomial of degree 15. In the latter choice, we determined the expansion coefficients through a least-square fit of a pseudowave function obtained from a Quantum Espresso calculation using the valence-only pseudopotential (this is shown as a green line in Figure 2a). Below we will compare the results for both choices of *f*(*r*).

The constant *N* in eq 17 is determined by requiring that Ψ̂_**k***n*_ is normalized to 1,
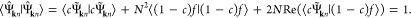
19

This is solved by
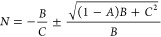
20where
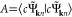
21
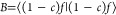
22
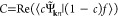
23

## 3 Results

In Figure 2, we compare the radial components of Ψ̂_**k***n*_(*r*,θ,φ) and Ψ̃_**k***n*_(*r*,θ,φ) for the lowest eigenvalue of bcc Na obtained at the Γ point. Note that we show in the figure results of pseudowave function and band dispersion for two choices of *f*(*r*) in eq 17: a constant and a 15-degree polynomial. Included for reference is also a pseudowave function obtained from a Quantum Espresso calculation (from which one choice of *f*(*r*) was obtained). In this calculation, we set the muffin-tin radius *R*_*MT*_ = 3.285 au, which is approximately 95% of the touching radius between two nearest Na atoms, and used *R*_0_ = 0.55*R*_*MT*_ and *R*_1_ = 0.75*R*_*MT*_ for the constant *f*(*r*) choice, and *R*_0_ = 0.75*R*_*MT*_ and *R*_1_ = 0.9*R*_*MT*_ for the polynomial choice. The all-electron calculation that is used to evaluate the pseudowave function uses the local density approximation (LDA).^40,41^ As LMTO basis vectors, we used three 3s orbitals, three 3p orbitals and two 3d-orbitals in the muffin-tin spheres. In the interstitial region, the tails have kinetic energies of 0.3 Ry (for 3s-, 3p-, and 3d-orbitals), −2.3 Ry (for 3s-, 3p-, and 3d-orbitals) and −1.5 Ry (for 3s- and 3p-orbitals). The number of reciprocal lattice vectors used to expand the wave function were 11 × 11 × 11 (corresponding to an energy cutoff of ∼18 Ry). This value of the cutoff energy is much smaller than the one used for standard pseudopotential calculations and still produces results in good agreement with those of different standard pseudopotentials. As in Figure 1, the experimental^27^ lattice parameter of 4.225 Å was used.

It is clear from parts a and c of Figure 2 that Ψ̂_**k***n*_(*r*,θ,φ) and Ψ̃_**k***n*_(*r*,θ,φ) are identical in the interstitial region. These wave functions also coincide with the full all-electron wave function in the interstitial region (data not shown). From a detailed inspection of the radial components of Ψ̂_**k***n*_(*r*,θ,φ) and Ψ̃_**k***n*_(*r*,θ,φ), we note that the latter has a single node as opposed to the two nodes expected from a 3*s* state. The single node of the otherwise rather soft behavior of Ψ̃_**k***n*_(*r*,θ,φ) has to do with how the full-potential method of ref (6) represents the basis functions in the interstitial, in particular as a Fourier series (see eqs 6.38–6.42 of ref (6)). In order to obtain a pseudopotential that is as smooth as possible from eqs 6–10, we have made use of Ψ̂_**k***n*_(*r*,θ,φ) (in a Fourier representation) instead of Ψ̃_**k***n*_(*r*,θ,φ), since the former pseudowave function is by construction node-less inside the muffin-tin sphere (see Figure 2a). This choice leads to a much smoother pseudowave function that may be expressed with a minimum number of Fourier components. For this reason we have used the Fourier representation of Ψ̂_**k***n*_(*r*,θ,φ) in all the steps outlined in eqs 1–10, discussed in Section 2.

In parts a and c of Figure 2 (a and c), we note that depending on the choice of *f*(*r*), the behavior of Ψ̂_**k***n*_(*r*,θ,φ) inside the muffin-tin region is different. For the choice of a 15-degree polynomial for *f*(*r*), Ψ̂_**k***n*_(*r*,θ,φ) is by construction similar to the function obtained from a calculation based on Quantum Espresso (see Figure 2a). Although the behavior in the core region is explicitly constructed, a good match is not guaranteed from the outset. The freedom provided through the normalization constant *N* and the fact that the two regions (interstitial and muffin-tin) are stitched together with the help of the interpolation function *c*(*r*) rather than being matched at the muffin-tin boundary means that the two functions are allowed to differ. The near-perfect match in spite of this is a reassurance of the soundness of the interpolation procedure.

After having calculated a pseudopotential, using eqs 1–10 in combination with Ψ̂_**k***n*_(*r*,θ,φ), we calculate eigenvalues and eigenvectors from eq 5. This represents therefore the solution to a local pseudopotential, and the calculation was done using 1331 plane-wave components in the expansion of the wave function. In parts b and d of Figure 2, we show the resulting energy dispersion along the high-symmetry line, Γ – *H*, of the first Brillouin zone, for two different *in situ* pseudopotentials, one from a choice of *f*(*r*) as a constant and one from a choice of *f*(*r*) being a 15-degree polynomial. The two different *in situ* pseudopotential results are compared to the energy dispersion of an all-electron full-potential electronic structure method (see Figure 2, parts b and d) . We first note that the whole methodology described above is designed to yield the same eigenvalue and wave function in the interstitial region at the Γ point. Hence, it is gratifying that, for the Γ point, the eigenvalues from the *in situ* pseudopotential method and the all-electron full-potential method differ only in the sixth significant digit, irrespective of the choice of *f*(*r*) in eq 18. Furthermore, the energy dispersion is seen to agree very well between *in situ* pseudopotential theory and all-electron theory throughout the Brillouin zone. When comparing the two *in situ* pseudopotentials (evaluated from constant and polynomial choice of *f*(*r*)), we note that the agreement in band dispersion between all-electron theory and any *in situ* pseudopotential theory is surprisingly good. This holds true even when we set *f*(*r*) = 1. The latter has a pseudowave function that in the core region differs significantly from a traditional behavior (e.g., as seen from the results obtained from the Quantum Espresso calculation). The poorer choice of *f*(*r*) = 1 nevertheless results in an *in situ* pseudopotential that reproduces all-electron results throughout most of the Brillouin zone, demonstrating the robustness of our approach. The largest difference for the eigenvalues is observed at the zone boundary, which is not unexpected, considering that these states have crystal momentum farthest away from that state where the *in situ* pseudopotential was calculated (the Γ point).

**Figure 3:**
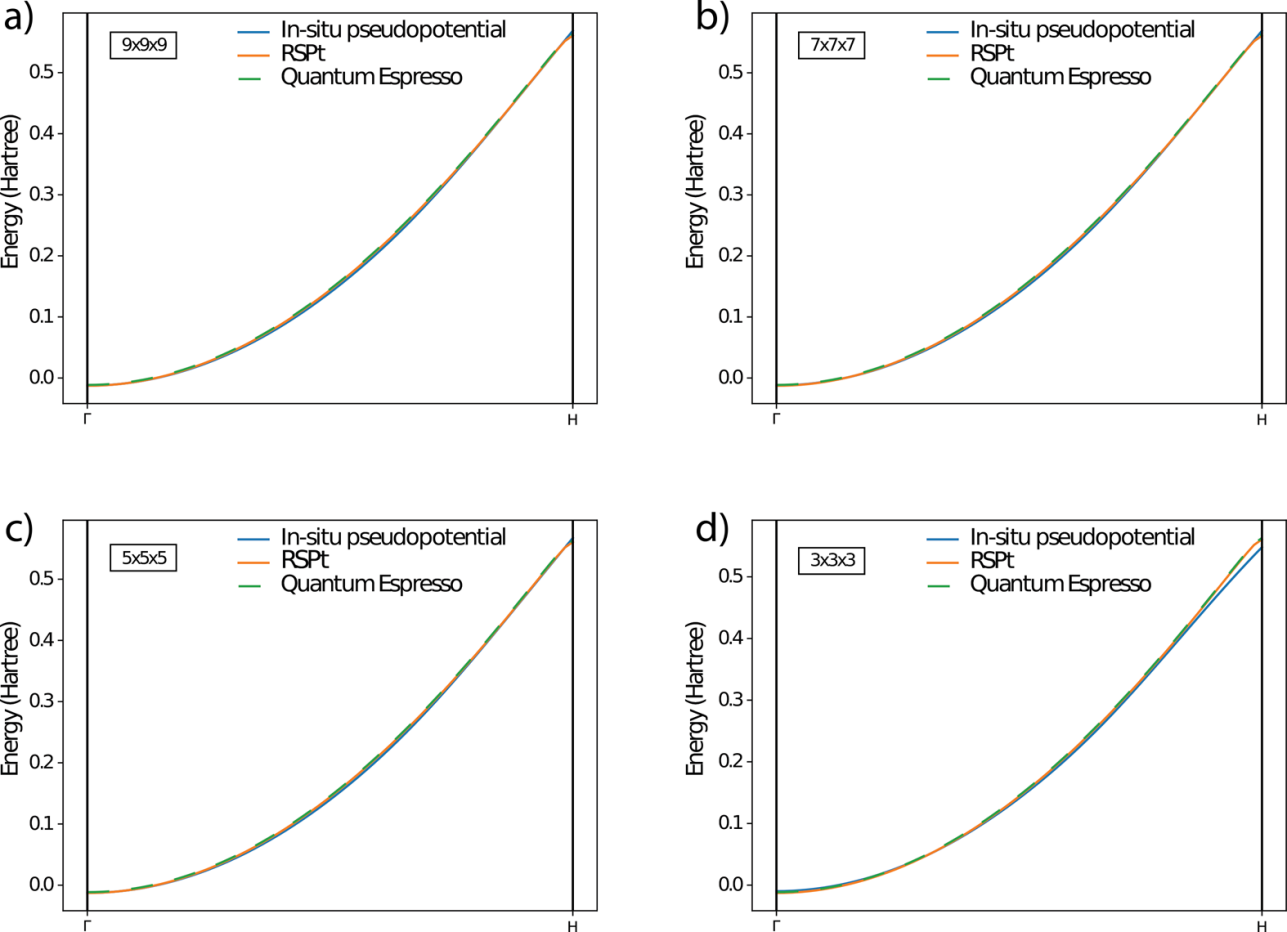
Calculated energy dispersion of the valence band states of bcc Na, along the Γ – *H* high symmetry direction of the first Brillouin zone. It is similar to the plot in panel b of Figure 2, except that the Fourier expansion of the pseudopotential is truncated to a smaller *N* × *N* × *N* mesh as specified in the inset of each panel. As in panel b of Figure 2, three types of methods are used to calculate the energy bands: the all-electron full potential method (orange), the *in situ* pseudopotential method as described of this paper (blue), and the standard pseudopotential method using a valence-only atomic pseudopotential (green). Here, *f*(*r*) is the 15-degree polynomial least-squares fitted to the radial pseudowave function calculated with Quantum Espresso using a valence-only pseudopotential (setting *R*_0_ = 0.75*R*_*MT*_ and *R*_1_ = 0.9*R*_*MT*_).

As a final point, we also investigate the effect of truncating the Fourier coefficients of the *in situ* pseudopotential to gauge its smoothness. In Figure 3, we show results of energy bands of bcc Na, when the *in situ* pseudopotential, being calculated from eq 10, has its higher Fourier components truncated. In practice this means keeping from the original *in situ* pseudopotential only components from a *N* × *N* × *N* mesh (that is smaller than the original mesh of 11 × 11 × 11). It is interesting to note from Figure 3 that good agreement between full-potential, all-electron theory and *in situ* pseudopotential theory can be achieved for all considered Fourier meshes except the very smallest one (3 × 3 × 3). This indicates that equal accuracy to all-electron theory can be achieved from an *in situ* pseudopotential theory that is represented by only 125 (5 × 5 × 5) plane waves.

## 4 Discussion and Conclusion

In this paper, we have demonstrated a proof-of-concept of how it is possible to calculate a pseudopotential from an all-electron, electronic structure method. For reference, in parts c and d of Figure 2, we also consider for a much cruder choice of core function, using *f*(*r*) = 1, *R*_0_ = 0.55*R*_*MT*_, and *R*_1_ = 0.75*R*_*MT*_. The agreement of the eigenvalues is in fact surprisingly good also in this case, even though the pseudowave function differs significantly from that of the Quantum Espresso in the core region. Technically, this amounts to solving the inverse Kohn–Sham equation for one or a few eigenvalues and eigenstates, which have been obtained from the all-electron theory. The method proposed here relies on replacing the rapidly oscillating part of an eigenstate close to the nucleus (in the muffin-tin sphere) with a smoother and much softer form, which allows for fast convergence in the expansion of the Fourier series. In principle, this method is not restricted to using the solid state as the reference electronic configuration and can be readily extended to molecular species or crystal surfaces. It also does not require the use of LMTOs as basis functions and is, for example, also suitable for the LAPW or LCAO basis set. It is also not mandatory to use the zone center to evaluate the *in situ* pseudopotential, other points of the Brillouin zone can be used as well, and it is possible to take averages of *in situ* pseudopotentials from several points, to get a final *in situ* pseudopotential to use for further studies.

In this work, we limit our discussion to the construction of the local components of the pseudopotentials. Its extension to the nonlocal components^19,20,25^ is natural and necessary in order to resolve higher lying energy bands, an effort which represents ongoing work. Even without the nonlocal components, the proposed *in situ* pseudopotential method reproduces energy dispersion of the 3s-like band state of bcc Na, with good accuracy throughout the Brillouin zone. The largest discrepancy between the *in situ* pseudopotential outlined here and results from an all-electron method is at the Brillouin zone boundary. This is expected since these zone-boundary states have an admixture of angular momentum characters that can only be properly described with the inclusion of the nonlocal components in the *in situ* pseudopotential. Furthermore, the crystal momentum of these states is the farthest away from the **k** point at which the *in situ* pseudopotential was calculated.

It is well-known that the transferability of an atomic pseudopotential can be systematically improved, by reducing *r*_*c*_ at the cost of greater computational cost.^19^ With the construction of the pseudopotential *in situ*, using the native state as the reference, this requirement of transferability can potentially be relaxed if an *in situ* pseudopotential is used. This may even allow for a larger pseudopotential radii cutoff for the same convergence, thereby reducing the number of Fourier components needed in the series expansion and a reduced computational cost. This method also automatically takes into account the nonlinear^35^ nature of the exchange and correlation interaction between the core and valence charge densities, which is important when a valence-only pseudopotential is used for an alkali metal like Na^35^ that only has one electron in the valence shell. In a typical calculation using atomic pseudopotentials, these interactions are first assumed to be linear before adding the nonlinear core contributions as a perturbative correction. Another benefit of generating the pseudopotential in the native solid-state environment is that basis-set convergence is already controlled at the level of the all-electron calculation. For example, if the LMTO basis set is used for the all-electron calculation (as in our case), convergence parameters will include the number of Fourier-components to match LMTOs as well as core-leakage that will indicate if certain semicore states have to be treated as valence states. Computational cost versus accuracy can then be optimized.

The methodology suggested here can also be extended to include spin-polarized calculations. One must then keep track of spin-indices of the all-electron generated eigenvalues and eigenvectors in the analysis presented in Section 2. Following the steps in the Method section that describe the pseudopotential generation, one could then obtain *in situ* pseudopotentials for spin-up states and spin-down states separately. After unscreening of these pseudopotentials (removing contributions from exchange and correlation of the electron gas, as well as the Hartree potential) one would obtain spin-dependent pseudopotentials that are able to accurately reproduce magnetic moments and spin-dependent information on all-electron theory. Spin–orbit effects may also be incorporated in the proposed *in situ* pseudopotentials, since the method outlined can be used for any spin–orbit calculated eigenstate. We also speculate that the *in situ* pseudopotential can be generalized, such that effects from a self-energy Σ^**k**^ (e.g., obtained from the *GW* approximation or the dynamical mean field theory) are incorporated in the pseudopotential. This could be achieved by associating ϵ̃_**k***n*_ with ϵ̃_**k***n*_ + ReΣ_**k**_ in eq 1. The steps outlined above represent an investigation that is underway. Finally, we note that a library of *in situ* pseudopotential-generating recipes can also be provided to supplement the library of pseudopotentials.
